# Intra-Patient Heterogeneity of Mechanical and Anatomical Properties in Thoracic Aortic Wall: An Ex Vivo Study Comparing Patients with Bicuspid and Tricuspid Aortic Valve Aortopathy

**DOI:** 10.3390/jcdd13010015

**Published:** 2025-12-28

**Authors:** Pasquale Totaro, Giulia Formenton, Martina Musto, Chiara Sciacca, Alessandro Caimi, Martina Schembri, Stefano Pelenghi, Ferdinando Auricchio

**Affiliations:** 1 Cardiac Surgery, IRCCS Foundation Hospital “San Matteo”, 27100 Pavia, Italy; s.pelenghi@smatteo.pv.it; 2 Department of Civil Engineering and Architecture, University of Pavia, 27100 Pavia, Italy; giulia.formenton01@universitadipavia.it (G.F.); alessandro.caimi@unipv.it (A.C.); martina.schembri01@universitadipavia.it (M.S.); auricchi@unipv.it (F.A.); 3 Department of Medicine, Surgical, Diagnostic and Pediatric Science, University of Pavia, 27100 Pavia, Italy; martina.musto01@universitadipavia.it (M.M.); chiara.sciacca@universitadipavia.it (C.S.)

**Keywords:** bicuspid aortic valve, ascending aorta, aortic aneurysm

## Abstract

Background: The ex vivo evaluation of the aortic wall aims to identify potential risk factors predictive of acute aortic syndrome. The comparison of aortic wall properties in patients with bicuspid aortic disease versus those with tricuspid aortic disease has been the subject of many studies. However, the heterogeneity of aortic wall characteristics in individual patients has never been thoroughly investigated. In this study, we focused on comparing the heterogeneity of aortic wall characteristics in patients with bicuspid (BAV) and tricuspid (TAV) aortic valve disease. Materials and Methods: Out of 113 patients enrolled in our cumulative study on the ex-vivo evaluation of the aortic wall, in patients with dilated ascending aorta, 56 patients with >3 specimens taken from the anterior wall were selected for the present study. The heterogeneity of anatomical characteristics (aortic wall thickness) was assessed by measuring the coefficient of variability (cV). In 35 patients, furthermore, mechanical (uniaxial ultimate stress–strain test) characteristics heterogeneity was also evaluated. Intra-patient mechanical and anatomical variability was then compared between the BAV and TAV groups. Results: Heterogeneity of aortic wall thickness was significantly less important compared to heterogeneity of mechanical properties: peak strain (Pstr *p* = 0.0042), peak stress (PS *p* = 0.001) and maximum elastic modulus (EM *p* = 0.001). Only EM heterogeneity was significantly reverse-correlated to patient’s age (*p* = 0.0005), and this correlation was peculiar for patients with BAV. In BAV patients, furthermore, age > 66 was associated with a significantly superior EM heterogeneity (*p* = 0.008). A direct comparison of anatomical and mechanical intra-patient variability between BAV and TAV groups, however, did not show significant differences. Discussion: Our study clearly demonstrates that the anatomical and mechanical characteristics of the aortic wall in patients with aortic dilation are not homogeneous. The heterogeneity of aortic wall thickness appears to be less significant than that of mechanical properties, thus confirming a limited correlation between anatomical and mechanical characteristics. The comparison between the BAV and TAV groups revealed limited peculiarities, further suggesting a preservation of the mechanical properties of the aortic wall in patients with bicuspid aortic disease and, therefore, without a peculiar mechanical properties-related increased risk of acute aortic syndrome.

## 1. Introduction

In recent decades, several studies have addressed the ex vivo assessment of the mechanical properties of the aortic wall in patients with dilated aorta undergoing surgical replacement of the ascending aorta, aiming to identify potential risk factors for acute aortic syndrome [1,2,3,4,5,6,7]. The translation of mechanical test results into clear recommendations for clinical practice has so far been limited mainly by two key unresolved issues. The first issue is related to the lack of a common standard protocol for the evaluation of the mechanical properties of the aortic wall. The different tests, parameters and threshold values used to date have made it very difficult to compare the results of different studies [1,2,3]. The second key issue is the relevance of the heterogeneity of anatomical and mechanical properties [8,9] depending on the location and type of stress applied. The heterogeneity of aortic wall characteristics provides clear evidence that estimating the risk of acute aortic events (i.e., rupture or dissection) based on a single measurement (maximum diameter of dilation) can be misleading. The maximum diameter of aortic dilatation, on the other hand, despite all the doubts raised in this regard, is still the main parameter suggested in the decision-making process in the current guidelines for the treatment of aortic diseases [10]. Understanding the real impact of intra-patient variability in the anatomical and mechanical properties of the aortic wall could therefore be the key issue to consider in the clinical translation of the results of mechanical tests on the aortic wall. We therefore designed this study with the primary endpoint of assessing the real impact of intra-patient variability in both anatomical and mechanical properties of the aortic wall in patients with ascending aortic dilatation. Furthermore, based on previous studies focusing on the mechanical properties of the aortic wall [11,12,13,14,15] and suggesting the presence of bicuspid aortic valve (BAV) as a predictive risk factor for early acute aortic syndrome, we compared, as a secondary endpoint, the heterogeneity of aortic wall characteristics in two groups of patients based on the presence of aortic disease with BAV or tricuspid aortic valve (TAV).

## 2. Materials and Methods

This study is a spin-off of a prospective study approved by the Institutional Ethics Committee of the IRCCS Fondazione Ospedale San Matteo (No. 20150005619—9 March 2015—amendment No. 20200019579—14 February 2020) and which received a ministerial grant. The overall study focuses on the comparative evaluation of ex vivo mechanical properties in aortic wall samples taken from patients with and without aortic dilation, and 187 patients have been enrolled to date. Depending on the extent of aortic dilation and the aortic cylinder taken during surgery, a variable number of tests are usually performed for each patient. Among patients undergoing elective surgery for ascending aortic dilation (n = 113), a subgroup of patients in whom multiple satisfactory tests were completed, allowing for an assessment of heterogeneity, was extracted and represents the cohort of the present study. Specifically, 56 patients (TAV 37 patients 66%, BAV 19 patients 34%) met the inclusion criteria for the analysis of intra-patient anatomical variability (>3 samples from the anterior region with the measurement of aortic wall thickness using a dedicated calliper) and 35 (TAV 22 patients 63%, BAV 13 patients 37%) met the inclusion criteria for the analysis of intra-patient variability of mechanical properties (>3 samples from the anterior region tested under circumferential force), as described in detail below. Aortic dilation was defined based on three different parameters: maximum aortic dilation (DMax), indexed dilation (ID) calculated as the ratio between the maximum diameter and the BSA index, and the area/height ratio of maximum dilation (A/Hr). In addition, two different phenotypes of aortic dilatation (i.e., the ascending aorta phenotype AAP and the root phenotype RP) were also considered. All samples were taken from the anterior region (greater curvature), which has been shown in our previous studies to be the most dilated and less uniform area of the aorta, suitable for several sample collections and heterogeneity assessments [16,17,18].

### 2.1. Mechanical Properties Analysis

**Figure 1:**
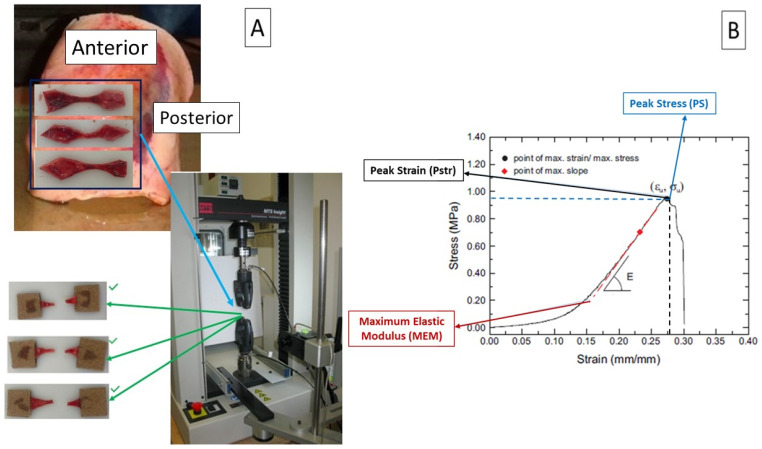
Summary of study protocol. (**A**) From the cylinder of aortic wall harvested at surgery, a variable number of specimens were obtained fom anterior (A) and posterior (P) region (**upper left**). Patients with at least 3 satisfactory tests from A region (**lower left**) were enrolled in the study analysis. (**B**) Mechanical properties analysis at rupture included three parameters: peak strain, peak stress and maximum elastic modulus (see text for explanation).

Mechanical tests of uniaxial tensile strength were performed on 342 fresh ex vivo samples within 48 h of collection, according to our previously published protocol [16,17]. In short, from the complete aortic cylinder harvested during surgery, it is possible to prepare several dog bone-shaped samples with a length-to-width ratio of at least 4:1. The samples are identified according to the region of the aortic wall, as defined in the previous paragraph (Figure 1A).

The number of samples obtained from each patient is related to the size of the aortic cylinder harvested and can vary from 3 to 13. The dog bone shape is characterized by a narrow central region identified by two markers. After careful measurement of the thickness with a dedicated calliper, uniaxial mechanical tests were performed using an MTS insight 10 kN testing system (MTS system corporation, Prairie, MN, USA). Each test was identified according to the region and direction of the aorta from which the sample was taken. Three different mechanical properties parameters (Figure 1B) were measured: peak strain (Pstr) as the maximum strain (ɛ_Max_) before specimen rupture (marker of aortic wall elasticity); peak stress (PS) as the maximum stress (σ_Max)_ before specimen rupture (marker of aortic wall strength); and maximum elastic modulus (EM) as the maximum slope (E_Max)_ of the stress/strain curve (marker of aortic wall stiffness).

### 2.2. Statistical Analysis

All data were recorded in a designed and approved database and statistical analysis was performed using Medcalc software (Medcalc 18.2.1; Acacialaan 22, 8400 Ostend-Belgium). Intra-patient (intra-observer) variability of the anatomical (aortic wall thickness) and mechanical parameters (ε_Max_, σ_Max_ and E_Max_) was evaluated by means of the coefficient of variation (cV = standard deviation/average × 100). Normal distribution for continuous variables was tested using the Kolmogorov–Smirnov test to optimize further statistical test choice. Comparative statistics was performed, using parametric (unequal variance, two tales *t*-test) or non-parametric test (Mann–Whitney for independent samples, Kruscal–Wallis) according to the results of the Kolmogorov–Smirnov test, to compare continuous variables of the two study groups. A comparison of categorical variables (gender, age > 66, maximum diameter > 52 mm, maximum indexed dilatation > 27.5 mm/m^2^, area/height > 11, phenotype of aortic dilatation, type of native aortic valve) was obtained with a chi-square analysis (fisher exact test when appropriate) or a Mann–Whitney test. A correlation Pearson’s coefficient or Spearman’s coefficient of rank correlation (rho) was used to evaluate linear correlation between numerical variables (patient’s age, maximum diameter of dilatation, area/height ratio, maximum index aortic dilatation) to intra-patient variability of anatomical and mechanical properties. Data were expressed as a mean ± sd or median/interquartile range according to normal distribution. Multiple logistic analysis was used to evaluate potential risk factors for cV above the 75th percentile.

## 3. Results

### 3.1. Patients’ Characteristics

Overall and comparative patient characteristics are summarized in Table 1.

The BAV group was characterized by significantly younger patients and with a significantly lower incidence of patients above 66 years. All parameters related to the extent of aortic dilation, on the other hand, were not significantly different in the two groups. The majority of patients (in both groups), finally, presented with an AAP of aortic dilation with no difference between groups.

### 3.2. Primary Endpoint: Analysis of Overall Intra-Patient Heterogeneity

**Figure 2:**
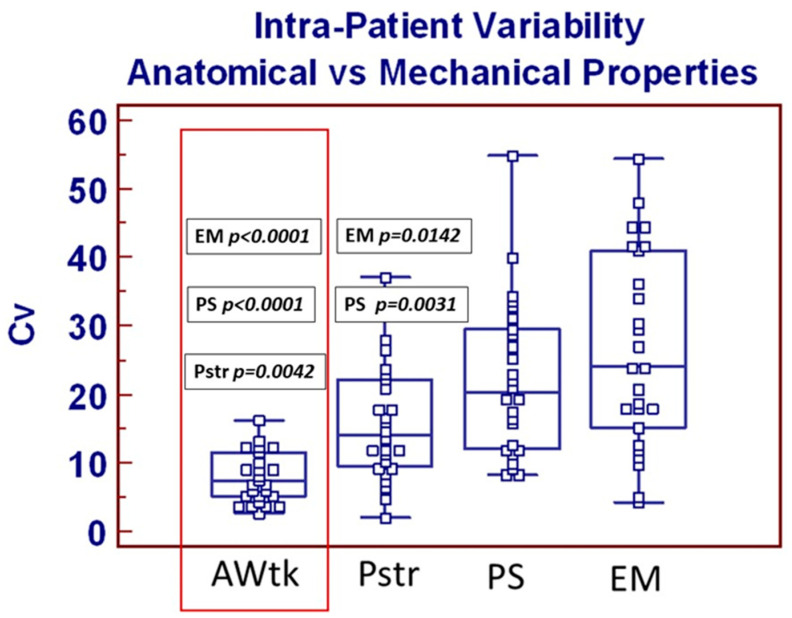
Summary and comparison of anatomical and mechanical properties intra-patient variability. Box-and-whiskers dot plots showing comparative analysis of intra-patient variability between the following: CV: Coefficient of variation; AWTk: Aortic wall thickness; Pstr: Peak strain; PS: Peak stress; EM: Maximum elastic modulus. *Borders of box: 1st and 3rd quartile, line in the box: median, whiskers: maximum and minimum values of non-outliers. All values higher/lower than the upper/lower inner/outer fence (3rd/1st quartile ± 1.5/3 IQR) are also plotted as outliers*.

An overall summary of anatomical and mechanical parameters is listed in Table A1. As previously showed by our groups [17,18], samples from BAV patients were significantly thinner compared to those from TAV patients. As far as mechanical properties are concerned, only peak strain was significantly reduced in samples from TAV patients. Overall intra-patient variability calculations are listed in Table A2. As summarized in Figure 2, anatomical intra-patient heterogeneity (specimen’s wall thickness) was significantly reduced compared to those of all three mechanical properties parameters (see red box).

**Figure 3:**
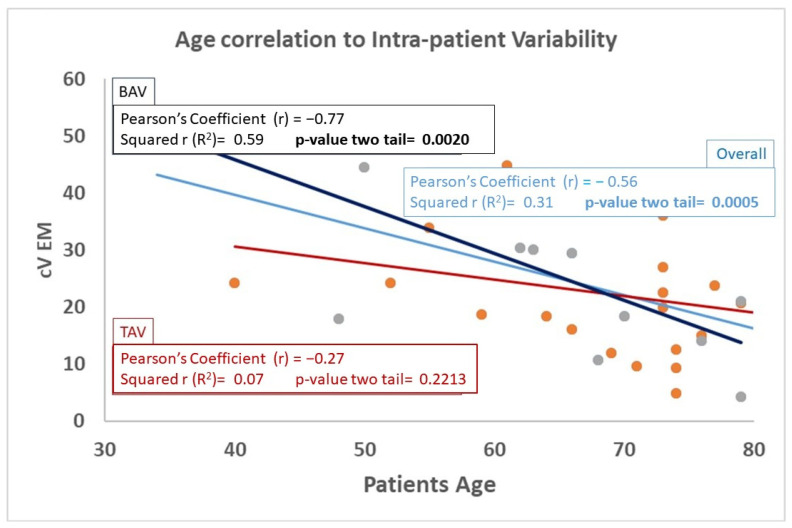
Significant correlation between intra-patient mechanical properties variability (cVEM: maximum elastic modulus coefficient of variation) and patient’s age in overall study population (light blue characters and line) and in the 2 subgroups according to the presence of bicuspid (BAV: bicuspid aortic valve—blackcharacters and line) or tricuspid (TAV: tricuspid aortic valve—red characters and line) native aortic valve.

Within mechanical parameters, furthermore, intra-patient variability of peek strain was significantly reduced compared to those of peek stress and maximum elastic modulus. Overall mechanical and anatomical characteristics correlation with all continuous variables were tested (see Table A2), and reverse correlation between patient’s age and aortic wall maximum elastic modulus was the only statistically significant correlation which resulted (see Figure 3). Finally, univariate analysis focused on the potential effect of categorical variables on anatomical and mechanical intra-patient heterogeneity, shown that only patients’ age > 66 was associated with increased intra-patient cV EM (*p* = 0.0015). Multivariate analysis including patients’ preoperative characteristics (listed in Appendix B) failed to show any risk factor for significantly increased intra-patient variability of three mechanical properties parameters according to previously defined criteria.

### 3.3. Secondary Endpoints: Comparison Between BAV and TAV Intra-Patient Heterogeneity

Moving on to the secondary endpoints and examining the comparative analysis between the two study groups, we first demonstrated that the significant correlation between patient age and intra-patient heterogeneity of aortic EM, illustrated above, was specific to the BAV group (Figure 3).

**Figure 4:**
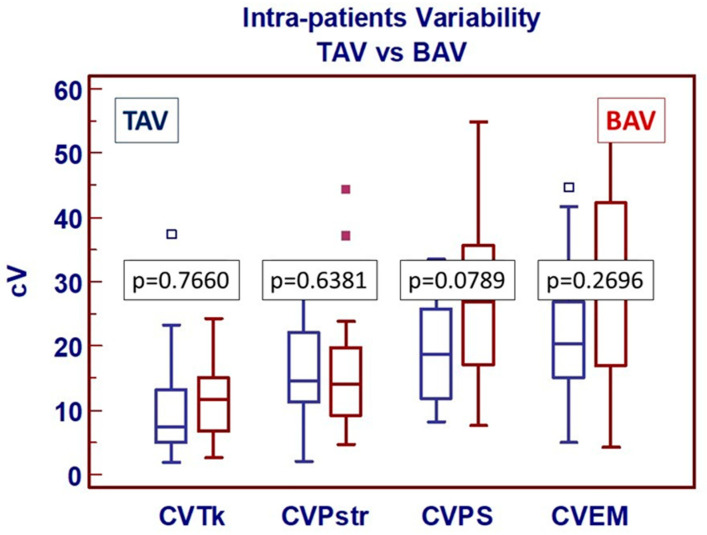
Comparative analysis of intra-patient variability according to the group of study. Box-and-whiskers dot plots showing comparative analysis (unpaired *t*-test) of intra-patient variability in patients with tricuspid aortic valve (TAV—blue bar) and bicuspid aortic valve (BAV—red bar). CVTk: Aortic wall thickness coefficient of variation; CVPstr: Peak strain coefficient of variation; CVPS: Peak stress coefficient of variation; CVEM: Maximum elastic modulus coefficient of variation. Borders of box: 1st and 3rd quartile, line in the box: median, whiskers: maximum and minimum values of non-outliers. All values higher/lower than the upper/lower inner/outer fence (3rd/1st quartile ± 1.5/3 IQR) are also plotted as outliers.

The univariate analysis focused on comparing continuous variables associated with intra-patient heterogeneity did not reveal any significant differences between the two groups (Figure 4).

**Figure 5:**
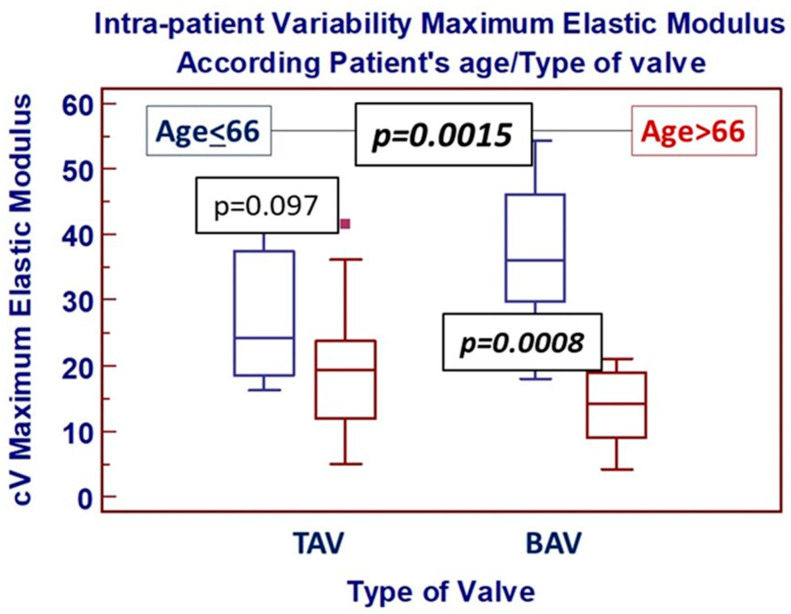
Intra-patient variability of maximum elastic modulus according to the type of native aortic valve and the age of patients (cut-off at 66 years old). Box-and-whiskers dot plots showing comparative analysis (unpaired *t*-test) of intra-patient variability of maximum elastic modulus in patients up to (blue bar) or older than (red bar) 66 years old and according to the presence of a tricuspid (TAV—left side) or bicuspid (BAV—right side) native aortic valve. *Borders of box: 1st and 3rd quartile, line in the box: median, whiskers: maximum and minimum values of non-outliers. All values higher/lower than the upper/lower inner/outer fence (3rd/1st quartile ± 1.5/3 IQR) are also plotted as outliers*.

The univariate analysis focused on comparing categorical variables confirmed, on the other hand, that the significance of the impact of patient age > 66 on EM heterogeneity was confirmed only in the BAV group (Figure 5).

## 4. Discussion

The aim of our study was to evaluate the real impact of intra-patient variability on the anatomical and mechanical properties of the aortic wall in patients with dilated ascending aorta. We hypothesized and designed our work based on the initial consideration that a more accurate understanding of the heterogeneity of aortic wall characteristics could be fundamental for translating the results of biomechanical tests into a clinical setting. Our study was therefore designed with two main endpoints: (1) to evaluate the real impact of intra-patient variability in anatomical and mechanical characteristics in patients with diseased aorta undergoing surgery, and (2) to evaluate potential significant differences in patients with bicuspid aortic valve compared to those with tricuspid aortic valve. The first important result, related to the primary endpoint, is the evidence that the overall intra-patient variability of aortic wall characteristics in patients with diseased aorta is a significant phenomenon, significantly more relevant for mechanical properties than for aortic wall thickness. This result seems to confirm, once again, that a reduction in aortic wall thickness observed in particular subgroups of patients (e.g., patients with bicuspid aortic valve) does not always correspond to a deterioration in mechanical properties [18,19]. Within the mechanical properties, resistance to rupture and deformation also appears to be less homogeneous than elasticity, as the coefficient of variation in maximum deformation is significantly lower than those of maximum stress and maximum elastic modulus. This aspect certainly deserves careful consideration, since the presence of a limited point of fragility could translate into a high local risk of aortic wall injury and could, therefore, represent the crucial correlation between mechanical characteristic parameters and clinical risk assessment. It is important to note that intra-patient variability of the aortic wall has so far been considered and reported in the literature as a form of heterogeneity in the comparison between different regions of the aorta (e.g., anterior/posterior, lateral/medial, superior/inferior) and, therefore, there is a lack of studies focusing on intra-patient variability of data within the same region of the aorta [12,19,20,21]. If evidence of regional heterogeneity has been considered proof of the inadequacy of a single parameter (maximum diameter of dilation) to accurately predict a high-risk condition of acute aortic syndrome, evidence of widespread heterogeneity even within the same region of the aorta (anterior wall) should be considered as further and significant support for this inadequacy. Identifying factors that could predict abnormal and developed intra-patient variability would be important for clinical interpretation, and so we tested a large number of parameters to explore this. First, we analyzed the impact of patient age, the only parameter that differed significantly between the two study groups. Based on our previous demonstration that patient age is directly correlated with increased aortic wall thickness [18], in this study, we found that it is also inversely correlated with intra-patient variability in terms of resistance to the deformation. From these data, we could therefore hypothesize that, in elderly patients with aortic dilatation, the aortic wall seems to undergo a physiological adaptation, becoming thicker and with a more homogeneous resistance to deformation. On the other hand, among all the preoperative characteristics of the patients tested, no significant impact on intra-patient variability was clearly demonstrated. In particular, the evidence that no parameter related to the extent of aortic dilation was significantly linked to the degree of intra-patient variability adds further doubt to the question of how a single “fixed” parameter can reflect the heterogeneous reality of aortic wall characteristics to the extent that it can actually be used as the sole criterion in the stratification of the risk of acute aortic complications in patients with ascending aortic dilatation [22]. Unfortunately, on the other hand, multiple logistic analysis failed to indicate a clear predictor for extremely increased variability in either aortic wall thickness or mechanical properties, thus necessitating further studies aiming to identify significant predictors of increased risk of acute aortic syndrome based on anatomical and mechanical properties heterogeneity.

Moving the focus of the discussion to our secondary endpoints, the comparison between BAV and TAV patients’ characteristics, we also showed some interesting findings. We have previously documented and commented [23] how the discrepancy in patient populations’ age, when comparing BAV and TAV patients undergoing aortic surgery, should not be considered as a limitation of the comparison, as it reflects the overall comparison of two subgroups of patients of different ages but similar stage of the disease (i.e., the surgical indication cut-off). Ultimately, we believe, therefore, that it would not be correct to analyse aortic wall properties in a matched population. The overall correlation between anatomical and mechanical properties heterogeneity, between BAV and TAV patients, however, did not reveal significant differences. These findings seem to indicate, once more, the lack of clear indicators for an increased frailty of aortic wall in patients with BAV aortopathy. Mechanical properties in BAV patients are, indeed, not impaired, as previous studies suggested, with a significantly better elasticity showed at rupture. The evidence of a not significant difference in terms of heterogeneity of mechanical properties in BAV patients seems, furthermore, to indicate that the uniformity of such characteristics, through the entire larger curvature of the aorta, is similar to TAV patients and, therefore, without an increased risk of an extremely frail area which could be at increased risk of rupture/dissection. The significant impact in BAV patients of age > 66 on increased heterogeneity of maximum elastic modulus, on the other hand, could indicate that, at this age, aortic disease is at an advanced step in BAV patients compared to TAV patients of the same age. In conclusion, the most relevant result of our study is the evidence that aortic wall characteristics in dilated and pathological aorta are variable not only according to a “regional” but also a local definition, and that clear risk factors related to the extent of such variability have not yet been identified. As previously demonstrated for the thoraco-abdominal aorta [24,25], we must therefore address the intrinsic variability of aortic wall characteristics also in the ascending aorta. The fundamental relevance of this issue is leading many teams to evaluate all potential non-invasive preoperative diagnostic tools [26,27,28,29] to easily investigate mechanical properties in vivo, also focusing on the assessment of intra-patient variability, which could certainly represent a key objective for the near future.

## 5. Conclusions

In patients with pathological and dilated ascending aorta, the anatomical and mechanical characteristics of the aortic wall are not uniform throughout its course. A better and more accurate understanding of the extent of this heterogeneity and, above all, the factors that can modify it could represent a significant step in the future identification of clinical risk factors for acute aortic syndromes. The presence of a BAV does not seem to be correlated to an increased anatomical or mechanical properties heterogeneity compared to TAV patients.

## 6. Limitations

The limitations of the present study are related, in accordance with a similar previous study, to the potential selection bias, as samples are all taken during surgery and therefore in diseased aorta. We are addressing such limitations by enrolling patients with non-diseased ascending aorta undergoing heart transplantation. This study also carries some further limitations mainly related to the unavailable option of a biaxial mechanical test [30,31,32], a methodology which we are also going to implement in the future.

## Figures and Tables

**Table 1:** Overall preoperative patient characteristics: BAV = bicuspid aortic valve; TAV = tricuspid aortic valve; BSA = body surface area; BMI = body mass index; AAP = ascending aorta phenotype; RP = root phenotype; ^§^ normal distribution according to Kolmogorov–Smirnov test. Percentage in brackets.

	Overall n = 56	BAV n = 19	TAV n = 37	*p*
**Gender**				
**Male**	34 (61)	13 (68)	21 (57)	0.5643
**Female**	22 (39)	6 (32)	16 (43)	
**Age (years) ^§^**				
**range**	28–85	28–79	32–85	
**mean ± sd**	64 ± 14	58 ± 16	68 ± 11	***0.0120***
**>66**	29 (52)	6 (31)	23 (62)	***0.0476***
**Weight (kg) ^§^**	75 ± 15	73 ± 12	77 ± 15	0.4723
**Height (m) ^§^**	1.69 ± 0.09	1.69 ± 0.09	1.70 ± 0.09	0.8827
**BSA (m^2^) ^§^**	1.88 ± 0.21	1.87 ± 0.23	1.878 ± 0.20	0.5736
**BMI (kg/m^2^) ^§^**	26 ± 4	25 ± 3	27 ± 4	0.2127
**Aortic Dilation (mm) ^§^**				
**range**	41–66	45–60	41–66	
**mean ± sd**	52 ± 5	52 ± 4	52 ± 6	0.7412
** ≥52**	22 (39)	7 (37)	14 (38)	0.8065
**Indexed Aortic Dilation (mm/m^2^) ^§^**				
**range**	22–38	23–37	22–38	
**mean ± sd**	27 ± 9	28 ± 4	27 ± 6	0.7228
**≥27**	32 (57)	10 (53)	22 (59)	0.7736
**Area/height ratio (mm^2^/m) ^§^**				
**range**	7–19	8–17	7–19	
**mean ± sd**	12.6 ± 2.4	12.6 ± 1.9	12.6 ± 2.6	0.8588
**≥11**	43 (77)	15 (79)	28 (75)	0.8915
**Phenotype of Aortic Dilatation**				
**AAP**	41 (73)	16 (84)	25 (68)	0.3377
**RP**	15 (37)	3 (16)	12 (32)	

## Data Availability

All data not included in the manuscript (or attached as appendix) are stored in the institutional database unavailable to the public due to privacy or ethical restrictions. Full data could be available, pending detailed and motivated request and consent from an institutional ethic committee to data diffusion.

## Abbreviations

The following abbreviations are used in this manuscript:

BAV	Bicuspid Aortic Valve
TAV	Tricuspid Aortic Valve
cV	Coefficient of Variation
Pstr	Peak Strain
PS	Peak Stress
EM	Maximum Elastic Modulus
Dmax	Maximum Aortic Dilation
ID	Indexed Dilation = Dmax/BSA (Body Surface Area)
A/Hr	Dilation Area/Height Ratio
AAP	Ascending Aorta Phenotype
RP	Root Phenotype

## Appendix A

**Table A1:** See Text for Explanation.

**Anatomical Parameter**	**Overall** **n = 56**	**BAV** **n = 37**	**TAV** **n = 19**	***p***
**Aortic Wall Thickness (mm)**				
range	0.7–3.9	0.7–2.8	0.9–3.9	
mean ± sd	1.8 ± 0.4	1.6 ± 0.3	1.8 ± 0.4	
median	1.7	1.6	1.7	***0.0008***
95% CI	1.7 to 1.8	1.6 to 1.7	1.7 to 1.8	
**Mechanical Parameters**	**Overall** **n = 35**	**BAV** **n = 13**	**TAV** **n = 22**	***p***
**Peak Strain (mm/mm)**				
range	0.01–0.79	0.07–0.67	0.01–0.79	
mean ± sd	0.31 ± 0.28	0.33 ± 0.34	0.30 ± 0.13	
median	0.28	0.34	0.27	***0.0012***
95% CI	0.27 to 0.30	0.28 to 0.37	0.25 to 0.29	
**Peak Stress (MPa)**				
range	0.11–6.54	0.22–3.78	0.11–6.54	
mean ± sd	1.47 ± 0.98	1.53 ± 0.87	1.45 ± 1.02	
median	1.26	1.37	1.23	0.1765
95% CI	1.17 to 1.36	1.16 to 1.54	1.13 to 1.34	
**Elastic Modulus (MPa/mm)**				
range	1.0–57.6	2.3–51.7	1.0–57.6	
mean ± sd	13.9 ± 9.4	16.1 ± 11.4	13.0 ± 8.3	
median	12.2	13.2	11.9	0.0566
95% CI	11.2 to 12.9	10.2 to 17.0	10.6 to 12.7	

**Table A2:** (See Text for Explanation) §: Normal Distribution.

**Coefficient of Variation**	**Overall** **n = 56**	**BAV** **n = 37**	**TAV** **n = 19**	***p***
**Aortic Wall Thickness §**				
range	2–37	3–24	2–37	
mean ± sd	10.6 ± 7.2	11 ± 6	10 ± 8	
median	9.08	11.6	7.45	0.7664
95% CI	7 to 11.9	6.6 to 15.3	5.5 to 11.2	
**Coefficient of Variation**	**Overall** **n = 35**	**BAV** **n = 13**	**TAV** **n = 22**	***p***
**Peak Strain §**				
range	2–44	5–44	2–28	
mean ± sd	16.2 ± 8.8	17.1 ± 11.6	15.6 ± 6.8	
median	14.09	14.09	14.5	0.6820
95% CI	11.8 to 18.0	8.3 to 25.3	11.4 to 21.8	
**Peak Stress §**				
range	8–54	15–54	8–33	
mean ± sd	22.4 ± 11.4	27.4 ± 13.6	19.5 ± 8.9	
median	21.02	26.9	18.7	0.0798
95% CI	14.6 to 27.9	15.5 to 40.5	11.9 to 25.6	
**Maximum Elastic Modulus §**				
range	4–54	4–54	5–45	
mean ± sd	24.5 ± 12.9	28.0 ± 15.4	23 ± 11	
median	21.07	29.4	20.3	0.2696
95% CI	18.1 to 29.8	13.7 to 44.8	15.3 to 26.3	

**Table A3:** (See Text for Explanation).

Variable	Coefficient of Variability (cV)			
	Wall Thickness	Peak Strain	Peak Stress	Elastic Modulus
**Age**	r = 0.0424, *p* = 0.7664	r = 0.1136, *p* = 0.5289	r = 0.2882, *p* = 0.1038	***r = −0.5522***, ***p = 0.0005***
**Aortic Dilation**	r = 0.0944, *p* = 0.5100	r = 0.0000, *p* = 0.9999	r = 0.1821, *p* = 0.3103	r = 0.1755, *p* = 0.3286
**Indexed Aortic Dilation**	r = 0.2597, *p* = 0.0657	r = 0.0957, *p* = 0.5962	r = 0.2337, *p* = 0.1906	r = 0.0607, *p* = 0.7373
**Area/Height ratio**	r = 0.2240, *p* = 0.1140	r = 0.0746, *p* = 0.6801	r = 0.2505, *p* = 0.1596	r = 0.1639, *p* = 0.3622
**BSA**	r = 0.1664, *p* = 0.2433	r = 0.0470, *p* = 0.7949	r = 0.0256, *p* = 0.8875	r = 0.1122, *p* = 0.5342
**BMI**	r = 0.0854, *p* = 0.5514	r = 0.1607, *p* = 0.3716	r = 0.0039, *p* = 0.9829	r = 0.0370, *p* = 0.8379

## Appendix B

**Table A4:** Variables included in multivariate analysis. (entered the model if *p* < 0.2 at univariate analysis).

Male (Female) gender
Patients’ Age > 66
AAP (RP) phenotype of aortic dilatation
BAV (TAV) type of native aortic valve
BMI > 28.6 (<23.6) kg/m^2^
BSA > 2.0 (<1.7) m^2^
Weight > 85 (<66) kg
Height > 1.76 (<1.65) m
Maximum aortic diameter > 52mm
Maximum indexed dilatation > 27.5mm/m^2^
Area/height ratio > 11

AAP = ascending aorta phenotype, RP = root phenotype, BAV = bicuspid aortic valve, TAV = tricuspid aortic valve, BMI = body mass index, BSA = body surface area.
